# Research on body image cognition, social support and illness perception in breast cancer patients with different surgical methods

**DOI:** 10.3389/fpsyg.2022.931679

**Published:** 2022-09-23

**Authors:** Yuhan Liu, Wanli Liu, Yinglu Ma, Xiaoyue Yang, Han Zhou, Tingting Zhang, Shuhong Shao

**Affiliations:** ^1^Department of Medical Psychology, Binzhou Medical University, Yantai, Shandong, China; ^2^The First School of Clinical Medicine of Binzhou Medical University, Binzhou, Shandong, China; ^3^Department of Breast Surgery, Binzhou Medical University Hospital, Binzhou, Shandong, China; ^4^Department of Psychology, Binzhou Medical University Hospital, Binzhou, Shandong, China

**Keywords:** breast cancer, illness perception, social support, breast conservation, body image cognition

## Abstract

In parallel with the rapid rise in breast cancer incidence, there is also a noticeable rise in the number of patients who experience persistent negative body image cognition after breast cancer surgery. This study aimed to explore the differences in illness perception, social support, and body image cognition among breast cancer patients with different surgical methods, and the correlation, regression, and mediation among the three variables. The Brief Illness Perception Questionnaire (BIPQ), the Social Support Rating Scale (SSRS) and the Body Image Cognition after Breast Cancer Questionnaire (BIBCQ) were used to evaluate breast cancer patients’ illness perception, social support and body image cognition. Data analyses were performed by descriptive statistics, independent samples *t*-test, analysis of variance (ANOVA), Pearson correlation, and linear regression. The mediation was explored by the PROCESS V3.3. The study found that breast cancer patients undergoing radical mastectomy (RM) and modified radical mastectomy (MRM) demonstrated more negative illness perception, body image cognition, and lower social support compared with the patients receiving nipple-sparing mastectomy (NSM; *p* < 0.05). The subscale cognitive representation (CR) of BIPQ was strongly positively correlated with BIBCQ (*p* < 0.05). Illness perception positively predicted body image cognition (*p* < 0.01), while social support negatively predicted body image cognition. Social support partially mediated illness perception and body image cognition, exhibiting a positive role in post-operative body image cognition.

## Introduction

According to the World Cancer Report released by the WHO International Agency for Research on Cancer (IARC), breast cancer has become the world-leading cancer in 2020, and it ranks first among Chinese women, accounting for 17.1% of all female malignancies. Surgery is the first choice for patients with early-stage breast cancer (Burstein et al., 2021). At present, about 70% of breast cancer patients in China have received the modified radical mastectomy (MRM) named Auchincloss surgery. Compared with the radical mastectomy (RM) named Patey surgery that removes the pectoralis minor, MRM preserves the pectoralis minor. The MRM reduces the scope of surgery and the size of the scar. It also improves patients’ quality of life after surgery. The diseased breast is removed in the process of RM and MRM. The nipple-sparing mastectomy (NSM) preserves the appearance of the breast and guarantees adequate skin coverage for subsequent breast reconstruction (He et al., 2021). Though clinical trials have demonstrated that the survival outcomes have no significant difference in patients who received NSM or MRM (He et al., 2021), the prevalence of NSM in China remains lower than that in developed countries (Kummerow et al., 2015), especially in northern China (Yu et al., 2020).

Breasts, as an external organ and symbol of women, are subject to long-term external gazes and comments (Li et al., 2017). In the absence of breast tissue, patients’ femininity is undermined (Jabłoński et al., 2018), generating a sense of shame and affecting their body image cognition (Saeed et al., 2021). In 1935, Schilder pointed out that body image cognition was an impression of the body formed by self-observation and others’ evaluations (Schilder, 1999). Female breast cancer patients are particularly susceptible to body image disturbances in response to the breast deficiency caused by breast surgery, especially in the first year after the operation (Rosenberg et al., 2020). Although breast conservation is crucial to a patient’s post-operative body image cognition (Rosenberg et al., 2020), Chinese breast cancer patients are more concerned with the treatment outcomes (Yu et al., 2020). A complete cure of cancer is more important for them, thus they would rather suffer more psychological damage to guarantee the treatment effect. It can be supported by the fact that Chinese women preferred MRM and RM to treat breast cancer, even if they were eligible for breast conservation surgery (Kummerow et al., 2015). This is closely related to the high implementation rate of MRM in China (He et al., 2021). Hence, it is worth investigating the impact of mastectomy and breast conservation surgery on Chinese patients’ body image cognition. On the other hand, an emerging number of psychosocial oncology research mainly focused on the relationship between body image cognition and emotional reaction (Rosenberg et al., 2020; Zamanian et al., 2021), shame (Trindade et al., 2018) and quality of life (Aydın Sayılan and Demir, 2020; Rosenberg et al., 2020) whereas the influence of illness perception on body image cognition has rarely been explored.

Illness perception is defined as a patient’s understanding of the disease after combining professional advice, past experiences, family opinions, communications among patients and the various reactions after treatment. It is based on a theory proposed by Leventhal et al. in the 1980s (Leventhal and Contrada, 1998). When patients feel less control over the disease, they are inclined to believe that this disease will result in serious consequences. It incurs the incidence of negative attitude and poor treatment adherence in chronic diseases such as chronic kidney disease (CKD; Xiong et al., 2018), heart disease (Mosleh and Almalik, 2016) as well as tumors (Czerw et al., 2020). It is common for patients with advanced stages of cancer to suffer more physical and mental pain. However, the breast cancer perception varied across cultures and individual backgrounds. A Korean study found no significant relationship between illness perception and breast cancer stage. Since Asian women tended to avoid being informed of their own disease, they obtained less medical condition about themselves (Lee et al., 2019). In contrast to Dutch breast cancer patients, early-stage breast cancer patients in Japan were more pessimistic about their illness perception (van der Kloot et al., 2016). In addition, the post-operative appearance of breasts caused by different surgical methods influenced illness perception. There is a study showing that the breast reconstruction surgery restored the appearance of the breast and reduced the patient’s perceived sense of threat from breast cancer (Fanakidou et al., 2018). Therefore, it is necessary to investigate the breast cancer perception of patients who underwent different surgical methods in China.

Social support refers to a social network that provides any type of help to an individual in distress. Various individuals and social groups such as family members, friends, neighbors and colleagues can be sources of support (Hou et al., 2020). Patients diagnosed with cancer are under considerable stress (Eckerling et al., 2021). As a mediation factor of stress and multiple psychosomatic disorders (Usta, 2012), social support is considered to modulate the cognition of cancer patients (Zamanian et al., 2021). The previous literature documented some relationships between social support, illness perception and body image cognition. A negative correlation between social support and illness perception was revealed (Aydın Sayılan and Demir, 2020). Further analysis identified social support as an important mediator of psychological changes in breast cancer patients. In the mediation effect of social support, patients’ psychological resilience indirectly affected the quality of life (Zhang et al., 2017). Social support toward breast cancer patients developed them adaptive coping styles, which reduced the symptoms of depression and anxiety (Zamanian et al., 2021). Family is an indispensable unit of social support in Chinese culture. Post-operative women coped better with changes in body image cognition when they received positive social support from their families (Liu et al., 2021). Intimate support from spouses was also highly associated with the recovery of body image cognition (Jabłoński et al., 2018). Patients after breast conservation surgery had higher social support than the patients after MRM. This difference further affected the patient’s quality of life (Zhang et al., 2017). These evidences show that social support combined between hospital and family will enhance the effectiveness of breast cancer post-operative rehabilitation (Li et al., 2019).

Accordingly, the present study recruited patients diagnosed with breast cancer, with a first focus on the differences in illness perception, social support and body image cognition among patients receiving NSM, MRM, and RM. After different surgical procedures, we hypothesized that patients undergoing NSM would exhibit the most positive illness perception, body image cognition and the highest social support. Patients undergoing RM would report the most negative illness perception, body image cognition and the lowest social support. Secondly, the correlations among illness perception, social support and body image cognition would be investigated. In order to analyze the relationship between illness perception, social support and body image cognition, the surgical method and other demographic variables were set as control variables. The regression from illness perception to social support and body image cognition would be explored, also the regression from social support to body image cognition. In the mediation model, illness perception would be assumed as the independent variable, social support would be assumed as the mediator variable, and body image cognition would be assumed as the dependent variable. We hypothesized that social support would play a protective role in mediating the relationship between illness perception and body image cognition in post-operative breast cancer patients.

## Materials and methods

### Source of data

During October 2021 and January 2022, purposive sampling was undertaken from the medical recorded system in the oncology department and breast surgery department of Binzhou Medical University Hospital, using a convenience sampling strategy. The hospital was a governmental three-grade hospital located in Shandong Province, a province in eastern China with better economic conditions.

### Participants

The inclusion criteria for participation were that patients:

1. Female; 2. Were at least 18 years of age; 3. Had been diagnosed with breast cancer; 4. Received surgery for breast cancer; 5. Voluntarily participated in this study and gave written informed consent. The study excluded patients with concomitant diseases and active psychiatric disorders.

### Study design

Participants were provided with paper questionnaires including a demographic and clinical form, BIPQ, SSRS and BIBCQ. The author of the study, who is specialized in medical psychology, conducted face-to-face interviews in private rooms with the patients to understand their psychological situation. After explaining the purpose of this research to the participants, they were asked to fill out the questionnaires based on their most recent reality. Data collection took 20–30 min on average. During the interview, patients could withdraw if they so desired. The questionnaire scores were used to assess patients’ psychological status.

### Ethics approval

This study was performed in line with the principles of the Declaration of Helsinki. Approval was granted by the Ethics Committee of Binzhou Medical University (no. 2021-271). Informed consent was obtained from all individual participants included in the study.

### Measurements

#### Demographic and clinical form

The form was designed by the researchers. Demographic form included age, education level, marital status, residence, family income and occupation. Patients completed this part themselves. Clinical form included time since diagnosis, cancer stage, surgical method and current therapy. The current therapy referred to the therapy the patient was receiving at the time the investigator recruited them. The author filled out this section based on the patient’s medical records.

#### Illness perception

The Brief Illness Perception Questionnaire (BIPQ) consisted of nine items (Broadbent et al., 2006). The first eight items (consequences, timeline, personal control, treatment control, identity, concern, understanding, emotional response) were rated by a Ten-point Likert-type scale (0 = not at all, 10 = severely affected my life), ranging from 0 to 80. The last item (causes) was an open-ended question that asked patients to list three contributing factors to the disease. The eight items could be regrouped into three subscales: cognitive representation (consequences, timeline, personal control, treatment control, identity), emotional representation (concern and emotional response), and illness comprehensibility (understanding). To determine the extent to which the illness was considered harmful or harmless, an overall score was calculated. When the total scores were higher, the illness was perceived as more threatening.

#### Social support

The Social Support Rating Scale (SSRS) was used to measure the level of social support an individual received (Hou et al., 2020). It had 10 items in total, including three subscales that measured objective social support, subjective social support and availability. Objective social support (OSS) consisted of the material assistance an individual received, as well as the presence and participation of their social networks. Subjective social support (SSS) represented the emotional support experienced by the individual. It referred to the emotional experience and satisfaction that the individual was supported in society. Availability (AVL) meant the use of social resources by an individual. Respondents were assessed by a Four-point Likert-type scale (1 = poor social support to 4 = rich social support). The aggregate score indicated the degree of social support.

#### Body image cognition

The Body Image after the Breast Cancer Questionnaire (BIBCQ; Baxter et al., 2006) is a 53-item questionnaire that measured body image cognition along six dimensions: vulnerability (*VS*; to breast cancer), body stigma (BSS; impairment of femininity and attractiveness), limitations (LS; daily functioning), body concerns (BCS; satisfaction with body image), transparency (TS; appearance after breast surgery) and arm concerns (ACS; any discomfort in the arm) in the context of breast cancer. Both questions 1–28 (1 = strongly disagree to 5 = strongly agree) and questions 29–53 (1 = never to 5 = always) were scored on a Five-point Likert-type scale. Negative responses to body image cognition contributed to higher scores.

### Data analysis

The Statistical Package for Social Sciences (SPSS) 20.0 was used for data analysis. Measurement data was described as mean ± standard deviation. To examine the effects of different surgical methods and demographic-clinical characteristics on social support, illness perception, and body image cognition, independent samples *t*-tests and ANOVA were used. The effects of the variables on each other were examined by means of Pearson correlation. A *p*-value of 0.05 was considered statistically significant.

Taking illness perception as the independent variable (X), social support as the mediator variable (M), and body image cognition as the dependent variable (Y), the mediation analysis was based on Model 4 in SPSS PROCESS V3.3(by Andrew F. Hayes; Igartua and Hayes, 2021) with bootstrapping (5,000 bootstrapped samples) using 95% confidence intervals (CI). Both demographic and clinical factors involved in this study were set as covariates. The effect was termed significant when the 95% CI excluded 0. The bootstrap test was devised into three steps. First, test the significance of a for model X ➛ M. Second, test the significance of b, c′ for model X ➛ Y and M ➛ Y. Third, if c′ was not significant, then there was a complete mediation. If c′, a and b were significant, plus c′ < c, there was a partial mediation. a*b/c denoted a mathematical interpretation for the mediation effect.

## Results

### Participants

A total of 173 breast cancer patients initially participated in the study, of which 157 eventually completed all questionnaires, with an effective response rate of 90.75%. Participants included patients who underwent RM (Patey operation), MRM (Auchincloss operation) and NSM (nipple-sparing mastectomy). The mean age of participants was 49.9 years (range = 31–76 years, SD = 8.65). More detailed demographic and clinical characteristics are presented in Table 1.

**Table 1:** Demographic and clinical characteristics (*n* = 157).

Variable		Number (%)
Age (years)	≤45	55 (35.03%)
	>45	102 (64.97%)
Education level	Below junior high school	116 (73.89%)
	High school	22 (14.01%)
	University or above	19 (12.10%)
Marital status	Married	140 (89.17%)
	Divorced/widowed	17 (10.69%)
Residence	Urban area	75 (47.77%)
	Rural area	82 (52.22%)
Family income	≤3,000	73 (46.50%)
	>3,000	84 (53.50%)
Occupation	Fixed job	37 (23.57%)
	No fixed job	120 (76.43%)
Time since diagnosis	<1 month	36 (22.93%)
	1–3 months	36 (22.93%)
	3–6 months	48 (30.57%)
	>6 months	37 (23.57%)
Cancer stage	I	9 (5.57%)
	II	110 (70.06%)
	III	35 (22.29%)
	IV	3 (1.19%)
Surgical method	NSM	21 (13.38%)
	MRM	112 (71.34%)
	RM	24 (15.29%)
Current therapy	Chemotherapy	117 (74.52%)
	Chemotherapy and others (radiation, targeted therapy or endocrine therapy)	40 (25.48%)

NSM, nipple-sparing mastectomy; MRM, Modified radical mastectomy (Auchincloss operation); RM, Radical mastectomy (Patey operation).

### The comparison of illness perception, social support, and body image cognition under different demographic and clinical characteristics

The scores of the SSRS were statistically different in age (*p* < 0.01), marital status (*p* < 0.01), residence (*p* < 0.05), family income (*p* < 0.01) and occupation (*p* < 0.01). The scores of the BIPQ differed significantly in age (*p* < 0.05), marital status (*p* < 0.01) and family income (*p* < 0.05). More data are given in Table 2.

**Table 2:** The differences in social support and illness perception among different sample characteristics groups (*n* = 157).

Variable	Social support			Illness perception		
	Mean (*x ± s*)	*t/F*	*p*	Mean (*x ± s*)	*t/F*	*p*
Age		4.33^**^	<0.001		2.32^*^	0.022
≤45	33.82 ± 7.07			32.60 ± 9.90		
>45	28.95 ± 6.52			36.42 ± 9.84		
Education level		1.05	0.352		0.93	0.396
Below junior high school	30.24 ± 7.13			35.66 ± 10.47		
High school	31.05 ± 7.40			34.32 ± 7.99		
University or above	32.74 ± 6.38			32.42 ± 9.00		
Marital status		3.99^**^	<0.001		4.48^**^	<0.001
Married	31.41 ± 6.56			33.91 ± 9.51		
Divorced/widowed	24.47 ± 8.38			44.76 ± 8.76		
Residence		2.35^*^	0.020		1.09	0.277
Urban area	32.03 ± 7.44			34.17 ± 10.42		
Rural area	29.40 ± 6.55			35.91 ± 9.58		
Family income		3.63^**^	<0.001		2.00^*^	0.047
≤3,000	28.53 ± 6.29			36.78 ± 9.38		
>3,000	32.50 ± 7.26			33.61 ± 10.34		
Occupation		3.01^**^	0.003		1.53	0.128
Fixed job	33.65 ± 8.25			32.89 ± 9.67		
Unfixed job	29.73 ± 6.45			35.76 ± 10.04		
Time since diagnosis		0.69	0.559		1.56	2.02
<1 month	30.22 ± 6.74			35.28 ± 8.12		
1–3 months	29.47 ± 7.76			37.64 ± 10.77		
3–6 months	31.60 ± 6.92			32.92 ± 9.38		
>6 months	31.00 ± 7.05			35.22 ± 11.35		
Cancer stage		1.80	0.149		1.44	0.233
I	32.78 ± 6.69			32.89 ± 7.15		
II	30.77 ± 7.35			35.08 ± 10.10		
III	30.49 ± 6.24			34.69 ± 9.99		
IV	22.00 ± 1.73			46.33 ± 11.37		
Current therapy		1.13	0.260		0.92	0.358
Chemotherapy	30.28 ± 7.21			35.51 ± 9.86		
Chemotherapy and others (radiation, targeted therapy or endocrine therapy)	31.75 ± 6.68			33.28 ± 7.21		

* *p* < 0.05;

** *p* < 0.01.

### Body image cognition, social support and illness perception in patients with different surgical methods

As shown in Table 3, compared with the MRM group and the RM group, the NSM group scored lower on three subscales of the BIBCQ: body stigma (BSS; *p* < 0.01), body concerns (BCS; *p* < 0.01), transparency (TS; *p* < 0.01), and the total score (*p* < 0.01). The NSM group had the highest score of the SSRS (*p* < 0.01) and the lowest score of the BIPQ (*p* < 0.05).

**Table 3:** The body image cognition, social support and illness perception scores after different surgical methods (*n* = 157).

	NSM① (*n* = 21)	MRM②(*n* = 112)	RM③ (*n* = 24)	*F*	*p*	LSD
Body image cognition	93.57 ± 17.04	111.62 ± 20.20	120.21 ± 22.02	10.41^**^	<0.001	① < ② < ③
Vulnerability	24.86 ± 7.68	26.98 ± 8.00	27.13 ± 7.94	0.67	0.514	/
Body stigma	21.29 ± 6.57	28.14 ± 7.61	32.79 ± 9.51	12.31^**^	<0.001	① < ② < ③
Limitations	18.24 ± 3.10	19.91 ± 4.39	21.04 ± 5.30	2.30	0.104	/
Body concerns	12.52 ± 2.89	15.09 ± 3.34	15.21 ± 3.72	5.44^**^	0.005	① < ② < ③
Transparency	9.71 ± 4.20	13.35 ± 3.52	15.00 ± 3.60	12.69^**^	<0.001	① < ② < ③
Arm concerns	6.95 ± 2.31	8.14 ± 2.86	9.04 ± 2.96	3.11^*^	0.047	① < ② < ③
Social support	35.38 ± 6.51	30.11 ± 7.12	29.08 ± 5.83	5.95^**^	0.003	③ < ② < ①
Objective support	9.76 ± 1.30	8.32 ± 1.71	8.72 ± 1.98	6.32^**^	0.002	③ < ② < ①
Subjective support	18.24 ± 4.72	15.13 ± 4.32	14.63 ± 3.84	5.14^**^	0.007	③ < ② < ①
Availability	7.38 ± 2.50	6.65 ± 2.82	5.71 ± 2.00	2.26	0.108	/
Illness perception	31.05 ± 7.87	34.96 ± 9.92	39.17 ± 10.83	3.86^*^	0.023	① < ② < ③
Cognitive representation	18.14 ± 4.59	20.81 ± 6.35	22.83 ± 7.17	3.13^*^	0.046	① < ② < ③
Emotional representation	9.52 ± 4.91	9.96 ± 5.04	10.63 ± 4.95	0.29	0.753	/
Illness comprehensibility	3.38 ± 2.71	4.19 ± 2.98	5.71 ± 3.18	3.76^*^	0.025	① < ② < ③

* *p* < 0.05;

** *p* < 0.01.

### Correlations between illness perception, social support and body image cognition

**Figure 1:**
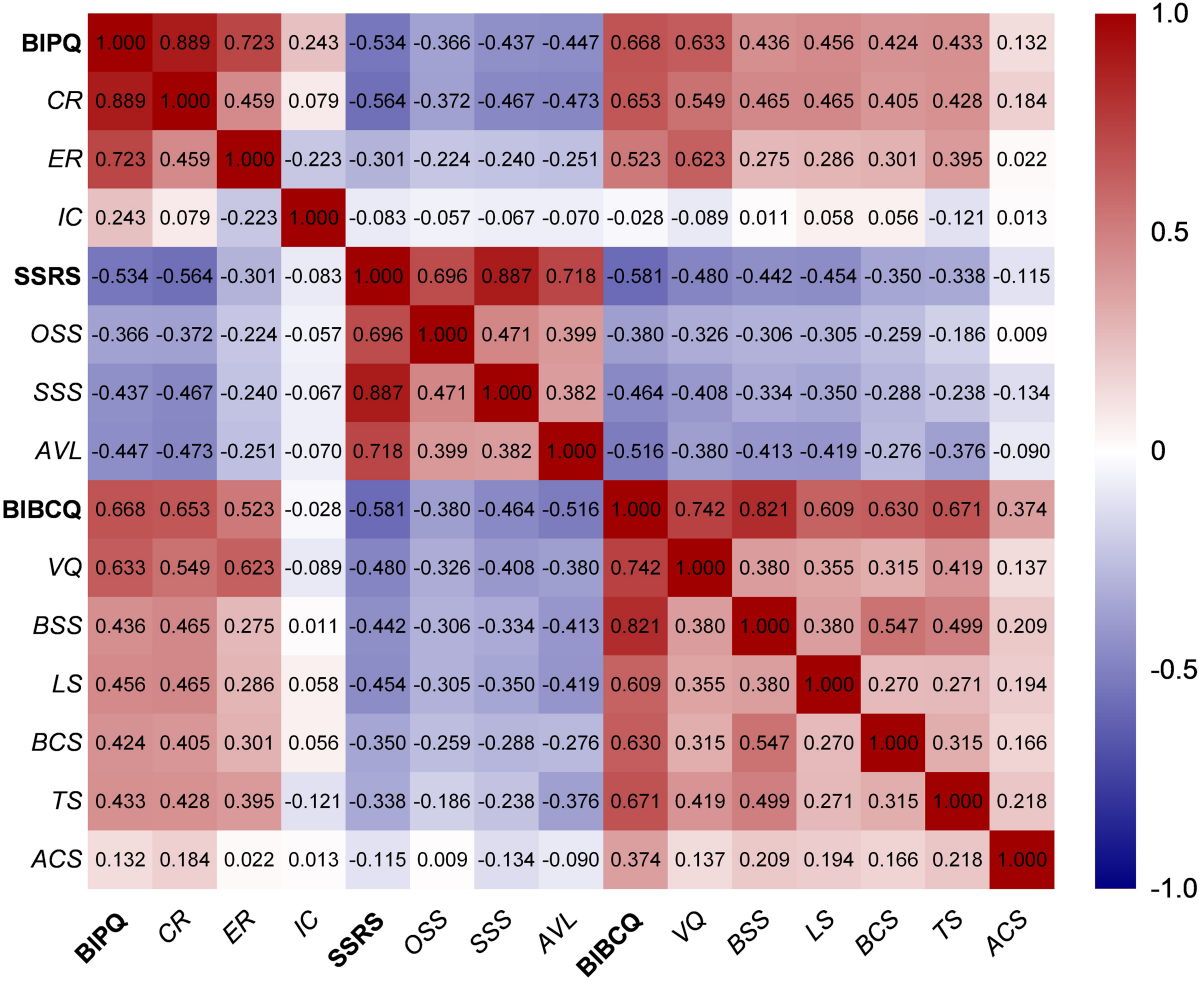
Heatmap of Pearson correlations in BIPQ, SSRS and BIBCQ. Figure represents the coefficients of the correlation analysis of illness perception, social support, and body image cognition. BIPQ, the brief illness perception questionnaire; CR, cognitive representation; ER, emotional representation; IC, illness comprehensibility; SSRS, social support rating scale; OSS, objective social support; AVL, availability; BIBCQ, the body image after the breast cancer questionnaire; VQ, vulnerability scale; BSS, body stigma scale; LS, limitation scale; BCS, body concerns scale; TS, transparency scale; ACS, arm concerns scale.

There was a significant positive correlation between the BIPQ and the BIBCQ (*p* < 0.01) as well as their subscales. Both BIPQ and BIBCQ were negatively correlated with the SSRS and its subscales (*p* < 0.05). Other correlation coefficients are shown in Figure 1.

### Regressions between illness perception, social support and body image cognition

Age, education level, marital status, residence, family income, occupation, time of diagnosis, cancer stage, surgical method and current therapy were set as covariate. The regression analysis results are shown in Table 4. It revealed that illness perception positively predicted body image cognition (*β* = 0.582, *p* < 0.01), 51.7% of body image cognition could be effectively explained by illness perception. Illness perception negatively predicted social support (*β* = −0.401, *p* < 0.01), 38.8% of social support could be effectively explained by illness perception. Illness perception and social support negatively predicted body image cognition (*β* = −0.304, *p* < 0.01), 57.3% of body image cognition could be effectively explained by illness perception and social support.

**Table 4:** Regressions of illness perception, social support and body image cognition.

Dependent variable		Independent variable	*β*	*t*	*p*
Body image cognition (*R^2^* = 0.517)	c	Illness perception	0.582	9.06^**^	<0.001
Social support (*R^2^* = 0.388)	a	Illness perception	−0.401	−5.55^**^	<0.001
Body image cognition (*R^2^* = 0.573)	b	Social support	−0.304	−4.37^**^	<0.001
	c′	Illness perception	0.460	6.90^**^	<0.001

** *p* < 0.01.

### The mediation effect of social support on the relationship between illness perception and body image cognition

**Figure 2:**
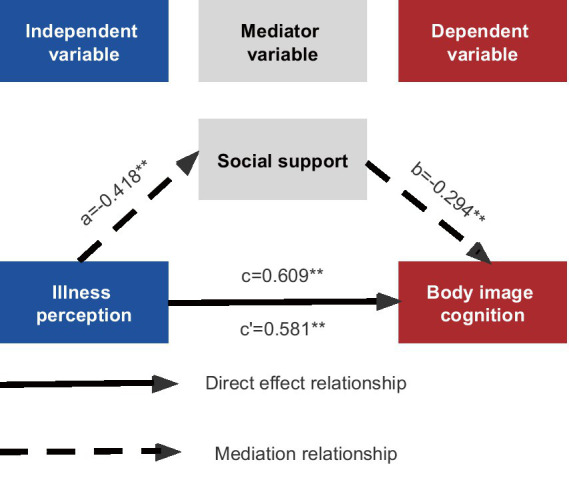
The diagram of the mediation effect of social support (^**^p < 0.01).

In the mediation model, illness perception was set as the predictor variable, body image cognition was set as the outcome variable, and social support was set as the mediator variable. Age, education level, marital status, residence, family income, occupation, time of diagnosis, cancer stage, surgical method and current therapy were set as covariate, with repeated sampling up to 5,000 times. The direct effect of illness perception on body image cognition was 0.581, with a 95% CI of [0.4553, 0.7067], excluding 0, indicating that the direct effect was significant. Illness perception influenced body image cognition through an indirect pathway partially mediated by social support, resulting in an indirect effect of 0.123, with a 95% CI of [0.0609, 0.2001], excluding 0, indicating that the indirect effect was significant (Table 5). The effect size of mediation was a*b/c = 20.14%. Figure 2 illustrates the mediation effect among illness perception, social support and body image cognition.

**Table 5:** Results of mediation effect.

Mediation		*β*	Boot SE	95%CI	
				LLCI	ULCI
Direct effect	Illness perception ➛ Social support	−0.418	0.073	−0.5613	−0.2744
	Social support ➛ Body image cognition	−0.294	0.069	−0.4293	−0.1581
	Illness perception ➛ Body image cognition	0.581	0.636	0.4553	0.7067
Indirect effect	Illness perception ➛ Social support ➛ Body image cognition	0.123	0.036	0.0609	0.2001

Boot SE, standard error; Boot LLCI, lower limit of 95%; Boot ULCL, upper limit of 95%.

## Discussion

In this study, patients undergoing NSM had more positive illness perception, body image cognition, and higher social support than patients undergoing MRM and RM. Illness perception negatively predicted patients’ social support while it positively predicted patients’ body image cognition. In the mediation effect, social support came out to be a partial mediator. In the presence of social support, the positive predictive effect of illness perception on body image perception is diminished.

As a threatening stressor, cancer and cancer related treatment interfere with patients’ cognitive function, resulting in hypersensitivity and sensory distortion (Eckerling et al., 2021). Surgical methods played a significant role in patients’ post-operative illness perception. For one thing, this study found that the cognitive representation (CR) in BIPQ had different outcomes in the groups of NSM, MRM and RM. Patients receiving NSM had lower CR scores in BIPQ, indicating that those patients perceived less threat from breast cancer. Different surgical methods, including the size of the incision, the preservation of the breast, and the degree of muscle and nerve damage, can affect the patient’s breast cancer perception after surgery. For anothor, there was no significant difference in the emotional representation (ER) in patients receiving NSM, MRM, and RM. It was explained that a diagnosis of breast cancer put patients’ health at risk (Liu et al., 2021), therefore, a pervasive negative impact will be exerted on the patients emotion regardless of the type of surgery.

Breasts are often regarded as an intimate part of a woman’s body. As a result of a breast surgery, women typically experience cosmetic impairment and body image concerns (Rosenberg et al., 2020). It is possible for patients to maintain their natural breast shape and structure after NSM (Zehra et al., 2020), thereby patients receiving NSM in this study experienced more positive body image cognition related to their appearance. On the other hand, an absent breast causing by MRM and RM seriously affected the breast’s appearance. Patients who underwent MRM or RM developed more negative body image cognition (Zehra et al., 2020), exhibiting the higher scores of body stigma (BSS), body concerns (BCS) and transparency (TS) in the BIBCQ. Since the patients were worried about that others would notice their absent breast after surgery, they felt more concerned about their bodies, thus the senses of shame and transparency increased. The most negative body image cognition was observed in patients after RM. The diseased breast was removed in the process of RM and MRM, but RM caused wider tumor dissection, more damage of the muscle and the nerve, and larger scars. These surgical injuries led to further deterioration of body image cognition.

There are two sources of explanation for the body image cognition after surgery. In the aftermath of breast surgery, patients’ self-consciousness about appearance was worse than expected, this discrepancy left them ashamed of their bodies (Rosenberg et al., 2020). In east Asia, inferiority and embarrassment imposed by social culture and prejudice were also responsible for body image cognitive impairment (Amini-Tehrani et al., 2021). The stigma of breast cancer has been rooted in cultural beliefs concerning bad luck, karma, and other traditional practices. Despite emigration to western countries, Chinese-American women were unable to reduce the stigmatization of breast cancer after surgery (Warmoth et al., 2017). In the long run, these changes led to the social isolation and poor quality of life in breast cancer patients (Liu et al., 2021), suggesting the importance of presurgical counseling to help patients understand body image changes rationally (Rosenberg et al., 2020), and to prevent self-devaluation through breast prosthesis, reconstruction and cognitive therapy (Zehra et al., 2020).

There was no significant difference in limitation scale (LS) in BIBCQ among patients undergoing NSM, MRM, and RM. This is because there is a standardization process for the daily care of patients in the three-grade hospitals (Lovelace et al., 2019), including the guidance of restoration of various physical functions. Professional nursing staff ensured the rehabilitation of patients’ physical functions during hospitalization. Nevertheless, the high scores of emotional representation (ER) in BIPQ and vulnerability (VL) in BIBCQ shows that there were negative emotions among breast cancer patients during their hospitalization, attributed to the lacking of high-quality cancer related psychological support (Li et al., 2019). Consequently, psychosocial support in addition to medical social support is rather crucial for breast cancer patients.

Patients undergoing NSM required higher medical costs and more time for the subsequent therapy (Yu et al., 2020). With more material support, these patients scored higher in the objective social support (OSS) in the SSRS. In addition, patients with NSM experienced little appearance change, leading to less social isolation of their social network (Liu et al., 2021). Therefore, they had a better emotional experience with their family members, friends and other groups. Since a stable spouse meant easier access to a reliable source of livelihood and ongoing emotional support (Ghizzani et al., 2018), married patients scored higher in SSRS. These reasons led to higher subjective social support (SSS) scores in patients after NSM. Among the patients undergoing different surgery, there was no significant difference in availability (AVL) in SSRS, indicating that all breast cancer patients had strong need for social support after surgery.

In the mediating effect model, the positive predictive effect of illness perception on body image cognition was lessened in the presence of social support. As such, it has been speculated that a sense of belonging to a social group or community may moderate the adverse cognition in breast cancer patients. Furthermore, there is psychobiological evidence that in groups with high social support, the neurohypophysis released more oxytocin to strengthen the bonds between individuals (Riem et al., 2021). Inhibition of the HPA axis reduced the stress-induced adverse health conditions (Iob et al., 2018). The mediating effect ratio of social support accounted for 20.14% of the total effect, implying other potential mediators such as hope (Hsu et al., 2021) and shame (Amini-Tehrani et al., 2021).

In this study, in comparison with the elderly patients, empathy and support were more easily accessible to younger patients because of the mismatch between age and the life-threatening disease (Champion et al., 2014). Thus the younger patients with early-onset cancer had higher social support and less negative illness perception. Rural patients face challenges such as financial problems, transportation barriers and limited opportunities for clinical trials (Charlton et al., 2015). The choices of treatment regimens and nutritional status of patients are largely determined by financial status (Sun et al., 2021), increasing a sense of control over breast cancer (Rosenberg et al., 2020). Similar studies have also found correlations between education deficiency, less income and lower social support (Coughlin, 2020).

This study showed that post-operative breast cancer patients exhibited lower social support. The cultural reluctance of Asian women and the suppression of negative emotions commonly prevent them from seeking psychological and emotional assistance (Warmoth et al., 2017). Inadequate patient-doctor communication and the cost of specialized psychotherapy are impediments to cancer psychosomatic recovery in China, calling for improvements in the doctor-patient relationship, from authorization to collaboration, as well as more health insurance coverage for cancer psychological care (Amini-Tehrani et al., 2021).

In conclusion, there is an urgent need for individualized treatment of cancer patients based on demographics and personality characteristics. If a patient is eligible, Chinese surgeons should recommend breast conservation for the breast cancer patient to ensure more positive illness perception and body image cognition and higher social support after surgery. Oncologists, psychiatrists, psychotherapists, social workers and relatives together constitute the social support network for breast cancer patients, which can accommodate patients’ postoperative cognitive change in a supportive manner.

## Limitations

Several limitations may affect the generalizability of this study. First, this study has limited sample cases. The subsequent study will collect more patients with different surgical methods. It will also be supplemented with the surgical methods that account for a smaller proportion in China, such as the breast conservation surgery, to verify the generalizability of the current study. Second, the causality of a patient’s psychological status cannot always be determined by cross-sectional studies. A wider selection of breast cancer patients will be studied in the future, including multicenter longitudinal studies in different hospitals and regions, to further investigate the influence of surgical methods on patients’ physical and mental recovery. Third, given that the participants in this study were all cancer patients, we minimized their fatigue in completing the questionnaire. Therefore, there are many more variables that could be incorporated in the future studies to explore their impact on the psychological status of breast cancer patients, such as medical insurance, breast cancer genotype, and patients past experience.

## Data availability statement

The raw data supporting the conclusions of this article will be made available by the authors, without undue reservation.

## Ethics statement

The studies involving human participants were reviewed and approved by Binzhou Medical University. The patients/participants provided their written informed consent to participate in this study.

## Author contributions

YL and SS contributed to the study’s conception and design and wrote the first draft of the manuscript. Material preparation, data collection and analysis were performed by YL, WL, YM, XY, and HZ. All authors commented on previous versions of the manuscript and read and approved the final manuscript.

## Funding

This study was funded by the National Natural Science Foundation of China (no. 81872332).

## Conflict of interest

The authors declare that the research was conducted in the absence of any commercial or financial relationships that could be construed as a potential conflict of interest.

## Publisher’s note

All claims expressed in this article are solely those of the authors and do not necessarily represent those of their affiliated organizations, or those of the publisher, the editors and the reviewers. Any product that may be evaluated in this article, or claim that may be made by its manufacturer, is not guaranteed or endorsed by the publisher.
